# Delivery in a vertical birth chair supported by freedom of movement during labor: A randomized control trial

**DOI:** 10.1515/med-2023-0633

**Published:** 2023-02-24

**Authors:** Dilek Hacıvelioğlu, Nurgül Güngör Tavşanlı, İrem Şenyuva, Funda Kosova

**Affiliations:** Midwifery, Banaz Family Health Center, Uşak, Turkey; Faculty Midwifery Department, Manisa Celal Bayar University Health Science, Manisa, Turkey; Department of Obstetrics and Gynecology, Uşak University Medical Faculty, Uşak, Turkey; Department of Medical Biochemistry, Manisa Celal Bayar University School of Health Services, Manisa, Turkey

**Keywords:** birth chair, delivery position, labor process

## Abstract

To evaluate the effect of delivery in a vertical birth chair (VBC) and traditional delivery table (DT) supported by women’s movement during labor on the labor process, fetal outcome, maternal hormone levels, birth comfort, and satisfaction. This randomized controlled trial was conducted with 1:1:1 allocation. Group 1: in the VBC in upright position, Group 2: on the DT in supine position, these groups supported by freedom of movement, control group: on the DT in supine position, labor in bed. The duration of second stage of labor was not different between the groups (*p* = 0.246). The occurrence of instrumental birth, episiotomy, and perineal laceration was also not different among the groups (*p* = 0.772, *p* = 0.953, and *p* = 0.124). The use of uterotonic was observed in control group (*p* = 0.001). 1 and 5 APGAR scores of newborns were not different in all groups (*p* = 0.121, *p* = 0.268). The lowest pain score was observed in Group 1 (*p* = 0.001). Birth comfort and satisfaction were higher in Group 1 (*p* = 0.001 and *p* = 0.001). Decreased postpartum prolactin levels and increased postpartum oxytocin levels were observed in the control and Group 1 (*p* = 0.004, *p* = 0.006). Freedom of movement during labor and delivery using VBC in upright position can play birth-promoting and supporting role. There were no negative effects on the fetal outcome.

## 1 Introduction

Freedom of movement during labor and delivery position affect motherhood, breastfeeding, and baby’s adaptation [1]. Maternal movements such as walking, squatting, leaning, etc. in the first stage of labor is critical for a favorable birth experience because it causes more uterine contractions, more maternal comfort, and less pain [2,3]. On the other hand, delivery positions (such as supine, recumbent, semi-recumbent, and upright) in the second stage of labor impact the labor progression, perineal injury, and maternal feeling [4–7].

Although supine position is associated with some advantages such as allowing the abdominal examination and aortic pressure assessment, it leads to pain and pelvic immobilization [7]. Upright position increases uterine blood flow, contractility, and pelvic outlet diameters, affecting the duration of labor [7]. Based on the advantages of the upright position, birth chairs (BCs), suitable for birth physiology, were designed such as AVE birthing bed and Birthing chairs-Magister Kebidanan (BC-MK15), and used throughout the world [7].

Endocrine system significantly affects the mother and baby during the delivery [8]. In a study on the evidence-based effects of the hormonal physiology on mothers and babies during birth, it was found that studies depending on the measurement of hormones should be prioritized to understand the beneficial or damaging effects of hormones in maternal care [9]. Oxytocin and prolactin (PRL) play a significant role during labor and in maintaining the health of the mother and baby; natural oxytocin increases uterine contraction, calmness, and reduce pain. Further, after delivery, oxytocin helps in lactation, while PRL plays role in the milk production and mother’s adaptations [1].

Although studies have been conducted to understand the effect of freedom of movement during labor and delivery positions (supine, traditional delivery table [DT] or upright, BCs) on the vaginal delivery, a study assessing the impact of the combination of maternal movement during labor and BC used during delivery is lacking. This study aimed to evaluate (1) the effect of use of vertical BC (VBC) [10] with upright position and traditional DT with supine position supported by women’s movement during labor on the labor process and birth outcomes, (2) birth comfort, satisfaction, and maternal hormones. The first null hypothesis was that there would be no significant differences between VBC and DT in terms of labor process and fetal outcomes, while the second null hypothesis was that there would be no significant differences between VBC and DT in terms of birth comfort, satisfaction, and maternal hormones.

## 2 Methods

### 2.1 Study design and participants

This randomized controlled trial included pregnant women who were 20–35 years of age, started the birth process at term, had no chronic illnesses, expected to give a normal birth without any obstetric risk, whose amniotic sac had not opened (mobility not hindered), and had not performed exercises during pregnancy. Pregnant women who had cesarean delivery due to indications or fetal distress during labor were excluded from the study.

### 2.2 Randomization

This was conducted between August 2019 and July 2020 in the maternity unit of a Usak Training and Research Hospital. Randomization was conducted according to the date of admission to the hospital for birth with 1:1:1 allocation. Pregnant women arriving on odd-numbered dates and meeting the criteria were included in the case groups, while those arriving on even-numbered dates were included in the control group. The groups were defined as follows: (1) traditional DT group consisted of women giving birth in the supine position, supported with freedom of movement and position during labor (*n* = 30), (2) VBC group consisted of women giving birth in the upright position, supported with freedom of movement and position during labor (*n* = 30), and (3) the control group, women with labor on the bed and delivery in the supine position (*n* = 30).

### 2.3 Data collection and intervention

After randomization, the case groups received instructions (approximately 30 min) under identical conditions using educational material showing movements, positions, and VBC (either visual or practical). In stage 1 of labor (after beginning, labor ends with full cervical dilation to 10 cm), the women were encouraged to move freely and take-up any position they want during the birth process, and included walking, squatting, sitting, leaning, and standing. They were also supported to make use of a pilates ball. When tired or monitoring or intervention was necessary, they were allowed to lie down for 10–15 min. At stage 2 of labor (completion of dilatation of the cervix to the delivery of the infant), women were brought to either VBC or the table for delivery. In the supine group, the birth occurred in the lithotomy position on the DT, with lying on the back, hips and knees bent, and legs supported with a stirrup. In the VBC group, the women’s back were brought to an angle of 60°. The women in the control group remained mostly on the bed during labor attached to electronic fetal monitorization, except when they used the toilet. The women in the control group did not receive any information on movements or positions.

In the VBC, the upright position depends on the angle made by the line joining the horizontal plane and the midpoints of the woman’s third and fifth lumbar vertebrae, and the angle should be more 45°. VBC is comfortable for pregnant women and suitable for the labor process and birth. It was created in Turkey in 2015 by Turkish researchers with the funding from the Scientific and Technological Research Council of Turkey [10–12].

The data collection instruments for all the groups in the study included visual analog scale (VAS) to evaluate pain (VAS, 0–10 cm; 0, no pain; 10, severe pain).

Birth comfort scale (BCS) was used at the beginning and the end of stage 1 of labor. The scale evaluated the physical, psychospiritual, and environmental factors. The point score is minimum 9 and maximum 45; a higher score indicates comfort [13].

Birth satisfaction scale revised (BSS-R) form was provided to mothers 20–24 h after birth. This scale evaluated the quality of care, stress during labor, and women’s individual features. The score ranges from 0 to 40, and high scores demonstrate high satisfaction [14].

Maternal blood oxytocin and PRL levels were analyzed using ELISA method as described previously [15]. The blood samples were collected at the time of admission to the maternity unit and 24 h after birth.

**Figure 1:**
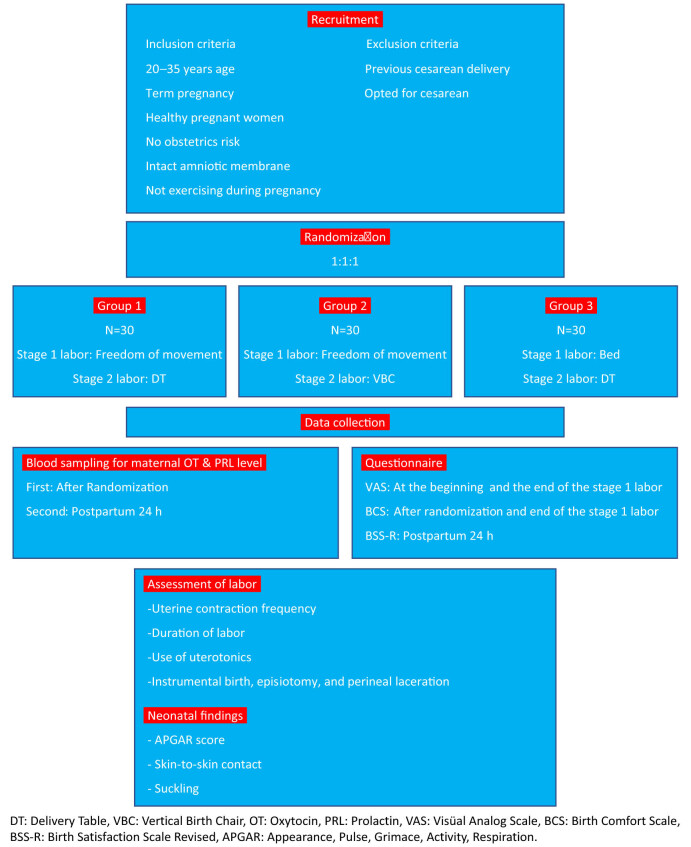
Flow chart.

Randomization, data collection, and interventions were carried out by two members of the research team (midwife; the first author) and obstetrician (the third author). Figure 1 shows the study flow-chart.

We compared the data related to the labor process, fetal outcome, birth satisfaction, comfort, and maternal hormone levels.


**Ethical considerations:** Permission for the research was obtained from the Health Sciences Ethics Committee of the Medical Faculty of Manisa Celal Bayar University (No. 10.07.2019/20.478.486) and Teaching and Research Hospital of Uşak University (No. 07.08.2019/45786011-612.01.99). The research was carried out in accordance with the Declaration of Helsinki. The women who agreed to take part in the study signed an Informed Voluntary Consent Form and an Approval Form for the Preservation of Biological Material for Educational and Research Purposes.

### 2.4 Statistical analysis

The program Number Cruncher Statistical System was used for statistical analysis. Kruskal–Wallis test and Dunn–Bonferroni test were used for comparisons between groups of more than two quantitative variables that did not show normal distribution. Wilcoxon signed-ranks test was used for in-group comparisons of quantitative variables that did not show normal distribution. Pearson chi-square test and Fisher–Freeman–Halton test were used to compare the qualitative data. Statistical significance was taken as *p* < 0.05.

Sample size calculation was performed with a computer application G*Power software (latest ver. 3.1.9.7). The calculation was performed by Cohen [16]. With the calculated effect size of 0.4, power 92%, and *α* error 0.05; the total sample size was 90.

## 3 Results

Ninety women were included in the study. Fifty percent of the women were primiparous and 50% were multiparous. No statistically significant difference was found between the groups in terms of gravida (*p* > 0.05). There were no differences between supine, VBC, and control group gestational weeks (39.33 ± 1.04, 39.02 ± 1.16, and 39.45 ± 1.07; *p* = 0.295). Table 1 shows the demographic characteristics of women.

**Table 1:** Demographic characteristics of women

	Group 1 (*n* = 30)	Group 2 (*n* = 30)	Group 3 (*n* = 30)	*p*
	Min–max (mean ± SD)	Min–max (mean ± SD)	Min–max (mean ± SD)	
Age	18–35 (25.60 ± 5.33)	19–34 (25.87 ± 4.55)	18–34 (26.70 ± 5.21)	0.679^a^
BMI (kg/m^2^)	16.6–31.3 (22.20 ± 4.22)	16–37.3 (22.91 ± 4.49)	17.9–36.7 (23.73 ± 4.03)	0.380^a^

BMI, body mass index; ^a^One-way ANOVA, ^b^Fisher–Freeman–Halton test.

### 3.1 Primary outcomes

#### 3.1.1 Assessment of labor

There was no significant difference in the duration of second stage of labor between the groups (*p* = 0.246). No significant differences were seen among groups in terms of instrumental birth, episiotomy, and perineal laceration (*p* = 0.772, *p* = 0.953, and *p* = 0.124). The need of uterotonic (in the second stage of labor) was observed significantly (*p* = 0.001) more in the control group than that in the DT and VBC groups. Table 2 shows the data on the assessment of labor.

**Table 2:** Assessment of labor

	**Group 1 (** *n* **= 30)**	**Group 2 (** *n* **= 30)**	**Control (** *n* **= 30)**	***p***
	**Min–max (mean ± SD)**	**Min–max (mean ± SD)**	**Min–max (mean ± SD)**	
Duration of second stage (min)	5–45 (20.5 ± 12.06)	5–35 (15 ± 8.41)	5–50 (18.23 ± 11.88)	0.246
Use of uterotonic (*n*, %)	2 (6.7)	4 (13.3)	14 (46.7)	**0.001**
Instrumental birth (*n*, %)	0 (0.0)	1 (3.3)	2 (6.7)	0.772
Episiotomy (*n*, %)	20 (66.7)	19 (63.3)	19 (63.3)	0.953
Perineal laceration (*n*, %)	0 (0.0)	4 (13.3)	4 (13.3)	0.124

#### 3.1.2 Neonatal finding

No significant differences were observed among groups in APGAR scores (at 1 and 5 min, *p* = 0.121, *p* = 0.268). Further, birth weight was also not different among the groups (*p* = 0.909) (Table 3).

**Table 3:** Neonatal findings

	**Group 1 (** ***n*** **= 30)**	**Group 2 (** ***n*** **= 30)**	**Control (** ***n*** **= 30)**	***p***
	**Min–max (mean ± SD)**	**Min–max (mean ± SD)**	**Min–max (mean ± SD)**	
1st min APGAR	8–9 (8.8 ± 0.41)	7–9 (8.73 ± 0.52)	7–9 (8.47 ± 0.73)	0.121
5th min APGAR	9–10 (9.8 ± 0.41)	8–10 (9.73 ± 0.52)	8–10 (9.57 ± 0.63)	0.268
Birth weight (g)	2,600–3,900 (3241.33 ± 371.05)	2,430–4,300 (3250.17 ± 436.17)	2,010–4,250 (3288.5 ± 515.64)	0.909

APGAR: appearance, pulse, grimace, activity, and respiration.

### 3.2 Secondary outcomes

#### 3.2.1 Birth comfort

While comparing the second VAS score, the lowest score was observed in the VBC group (*p* = 0.001). Second measurement of BCS score was also significantly higher (*p* = 0.001) in the VBC group. Similarly, VBC group showed a significantly (*p* = 0.001) higher BSS-R score. Table 4 shows the birth comfort results.

**Table 4:** Evaluation of VAS, BCS, and BSS-R scores

	**Group 1 (** ***n*** **= 30)**	**Group 2 (** ***n*** **= 30)**	**Control (** ***n*** **= 30)**	***p***
	**Min–max (mean ± SD)**	**Min–max (mean ± SD)**	**Min–max (mean ± SD)**	
**VAS**				
First measurement	0–7 (4.1 ± 2.06)	2–7 (4.27 ± 1.17)	1–7 (4 ± 1.62)	0.757^a^
Second measurement	7–10 (8.7 ± 0.75)	7–10 (8.53 ± 0.82)	7–10 (9.4 ± 0.67)	**0.001** ^b^
Difference	4.60 ± 1.81	4.27 ± 1.36	5.40 ± 1.69	
*p*	**0.001** ^c^	**0.001** ^c^	**0.001** ^c^	**0.034** ^b^
**BCS**				
First measurement	27–44 (38.63 ± 4.44)	25–45 (37 ± 4.89)	30–45 (38.17 ± 5.19)	0.339^b^
Second measurement	28–45 (42.07 ± 3.26)	42–45 (44.77 ± 0.63)	26–39 (33.57 ± 3.78)	**0.001** ^b^
Difference	3.43 ± 4.73	7.77 ± 4.57	−4.60 ± 3.55	
*p*	**0.001** ^c^	**0.001** ^c^	**0.001** ^c^	**0.001** ^b^
**BSS-R**	21–40 (31.03 ± 3.98)	22–37 (33.27 ± 3.56)	12–34 (21.37 ± 5.36)	**0.001** ^b^

VAS, visual analog scale; BCS, birth comfort scale; BSS-R, birth satisfaction score-revised. ^a^One-way ANOVA, ^b^Kruskal–Wallis test, ^c^Wilcoxon signed ranks test.

#### 3.2.2 Maternal hormones

Control group showed significantly (*p* = 0.004) decreased levels of postpartum PRL, while a significant (*p* = 0.006) increase in the postpartum oxytocin levels was observed in the VBC group. Maternal hormone levels are shown in Table 5.

**Table 5:** Comparison of biochemical results

	**Group 1 (** *n* **= 30)**	**Group 2 (** *n* **= 30)**	**Control (** *n* **= 30)**	***p*** ^**a**^
	**Min–max (mean ± SD)**	**Min–max (mean ± SD)**	**Min–max (mean ± SD)**	
**PRL (ng/mL)**				
Before	34.48–580.35 (167.2 ± 140.45)	27.98–943.9 (196.85 ± 266.2)	64.23–523 (148.01 ± 108.36)	0.165
After	35.56–490 (190.61 ± 148.94)	29.61–1037.49 (222.31 ± 298.76)	82.08–534.9 (150.32 ± 119.39)	0.420
Difference	−3.8 (−409 to 292.1)	−3.79 (−197.5 to 44.9)	15.69 (−239.7 to 49.2)	
*p* ^b^	0.382	0.078	**0.011**	**0.004** ^**a**^
**Oxytocin (pg/mL)**				
Before	11.9–188.62 (47.77 ± 43.76)	11.9–667.34 (100.35 ± 167.55)	13.22–150.37 (39.16 ± 32.61)	0.299
After	1.35–271.7 (68.08 ± 81.26)	26.41–829.56 (121.1 ± 205.99)	−1.29–163.56 (40.65 ± 37.4)	**0.034**
Difference	−1.32 (−230.8 to 38.2)	−15.83 (−162.2 to 30.3)	−6.59 (−26.3 to 34.3)	
*p* ^b^	0.264	**0.006**	0.784	0.146^a^

^a^Kruskal–Wallis test, ^b^Wilcoxon signed ranks test.

## 4 Discussion

Upright position in delivery leads to reduced pressure on the abdominal aorta and increases the blood flow in the uterine vessels, resulting in high uterine contractility [7]. This position increases the pelvic pressure (approximately 30–50 mmHg) more than that of the supine position. Hence, the pressure on the cervix stimulates Ferguson reflex and enables oxytocin secretion in the brain [7]. On the other hand, with the upright position, the fetal move toward the pelvic outlet, sagittal diameter of pelvic outlet increases, and transient umbilical cord compression decreases [6,7,17].

Many BCs have been developed based on the advantages associated with the upright position [7]. Although previously designed BCs allowed upright position, they were not comfortable during and after delivery, women were required to be moved to a different table for episiotomy repair [5,6]. Currently designed BCs are compatible for physiological process of delivery and provide maternal comfort, with option of choosing different positions such as upright position during delivery and supine position during the episiotomy repair [7]. It has been suggested that the table angle must be 45° or more for decreasing the abdominal aortic pressure, generating high uterine blood supply, and contractility; however, traditional tables do not allow such angles [17].

Several studies have been conducted to demonstrate the effectiveness of BC and DT during delivery. Fitriani et al. reported that in BC, duration of second stage of labor was significantly lower than that of the DT group (20.67 ± 1.02 and 26.06 ± 1.08, respectively, *p* = 0.001) in multiparous women [7]. Perineal damage generally prolong second stage of labor and is related to congestion and edema, and all of these may lead to injury [5]. Low rates of episiotomy and perineal damage have been associated with use of BC during delivery [18,19]. In a study with 55 primiparous women in the third trimester related to the use of BC and DT, they did not observe 1 and 5 min APGAR scores abnormality for the two groups, which were 8.3–8.5 and 9.3–9.4 (*p* > 0.05) [4].

On the other hand, in Cochrane review 9015, pregnant women were evaluated (without epidural anesthesia), and delivery in BC and supine position were compared. No differences were observed in stage 2 labor and APGAR scores; however, they detected less episiotomy rates and high second-degree tear in BC group [20]. Crowley et al. evaluated 1,250 nulliparous women (no epidural anesthesia); they also did not observe any positive effect of BC during delivery and on prevention of perineal trauma [21].

The primary null hypothesis of this study could be partially accepted. In fact, we did not detect abnormal APGAR scores in all of the babies and it can be explained by inclusion of healthy pregnant women without any obstetrics risk during the antenatal follow-up. Further, we did not observe less duration of second stage of labor in all the groups, which may be due to small number of patients and mixed parity type. We also did not observe any perineal injury or high episiotomy rate in all the groups, possibly due to the absence of prolonged second stage of labor.

The feeling of satisfaction with birth is a multi-dimensional perception and includes the physical and psychosocial experience of birth [22]. It is reported that satisfaction in the birth process was associated with greater comfort [13]. Upright position with BC, freedom of movement during labor positively affect the oxytocin release and this hormone crosses the blood–brain barrier and leads to calmness, maternal delivery comfort, and less vulnerability [1–4,7,19,23]. Also, increased oxytocin level leads to alleviate pain and milk ejection, while increased PRL levels impact milk production and mother’s adaptation [1]. There was limited research on BC and maternal comfort in the literature. Shannahan and Cottrell studied 55 primiparous women and compared the maternal comfort levels of BC and DT; they found that BC users were more satisfied (BC = 3.52, DT = 3.17, *p* = 0.037) [4]. Crowley et al. evaluated 1,250 nulliparous women, they stated that women thought the experience was “not too unpleasant” 51% of BC and 45% DT [21]. Maternal freedom of movement during labor was recommended as Evidence grade-A [17]. This is because, in freedom of movement, women can attain different postures (such as standing, kneeling, squatting, etc.), leading to fetal head impinging on the internal body of the clitoris and releasing oxytocin and pain relief [3,24]. Walking during the first stage of labor reduces the need for an epidural anesthesia in terms of pain relief, according to clear and significant evidence found in Cochrane study 5218, which assessed pregnant women (RR = 0.81, 95% CI = 0.66–0.99, nine studies, 2,107 women; random effects, T2 = 0.02, I2 = 61%) [25]. In their meta-analysis of 533 pregnant women, Grenvik et al. found that the birthing ball group experienced much less labor pain than the control group (MD = 1.70 points; 95% CI = –2.20 to –1.20) [26]. Pain associated with vaginal delivery can increase mother’s stress and it is a risk factors for delayed lactogenesis [27]. Fifty-one pregnant women were evaluated by Karakoyunlu et al. for their first stage of labor VAS score, active phase perceived stress scale (PSS), and breastfeeding scale. There was a moderately negative correlation between stress and breastfeeding (breastfeeding scale = 6.56 ± 1.64, PSS = 48.13 ± 4.09, and VAS score = 9.21 ± 1.04) [28]. The secondary null hypothesis of this study could be rejected, although supported by free maternal movements in both the groups. VBC group showed lower VAS score and high birth comfort and satisfaction than that of the DT group. It can be explained by the combined effects of being upright, the high mother oxytocin level, and experiencing less pain. On the other hand, required uterotonic treatment, higher VAS score, and lower PRL levels were seen in the control group. It may be explained by negative effect of inactivity on birthing physiology, inability of synthetic oxytocin to cross the blood–brain barrier, and it does not have the same relaxing effect as natural oxytocin [1].

According to Cochrane review; upright position in the second stage of labor and freedom of movement during the first stage of labor are considered advantageous for mothers and babies [20,25]. Also, some evidence-based care practices about nursing/midwifery education promote physiological birth: avoiding unnecessary induction of labor, allowing freedom of movement for the laboring woman, providing continuous labor support, and keeping mothers and babies together after birth without restrictions on breastfeeding [29]. In terms of continuous labor support and obstetrics care quality, one-to-one care is recommended by the current guidelines [30,31]. In this aspect, we showed positive effect of delivery in a VBC supported by freedom of movement during labor. Midwives and obstetricians should support the mother’s movement and consider the upright position by using VBC. Therefore, midwives’ numbers should be increased and their duties should be arranged in the delivery room [30]. To reveal the true benefits and risk of this approach further comprehensive studies are needed.

Limitations of the study: A specific position was not examined; therefore, the results cannot be generalized to a single position. Some factors other than movement and position may also have an effect on the results (such as prenatal perineal release applications and pushing at birth). Because of ethical reasons, second blood sample was collected at 24 h postpartum. Pulsatile oxytocin releases during breastfeeding and promotes PRL release; PRL remains above baseline during the 24 h period and throughout early postpartum weeks [32,33]. All patients received painkillers (short acting non-steroidal anti-inflammatory drugs) immediately after delivery only one time. The half-life of these drugs is <6 h [34], and we collected second blood sample after 24 h of delivery.

## 5 Conclusions

In this study, we demonstrated that freedom of movement during labor and delivery using BC in upright position positively affect the postpartum maternal hormone levels, decrease pain, increase birth comfort, and satisfaction, without any adverse effect on labor, birth, and fetal outcomes. Thus, freedom of movement combined with use of BC during labor can have satisfactory results during the vaginal birth.
